# Strictures on Dr. Grant's Latin Edition of His Essay on Yellow Fever

**Published:** 1806-11

**Authors:** Tho. Dancer

**Affiliations:** Author of the Jamaica Practice of Physic, or Medical Assistant


### Strictures on Dr. Grant's Latin Edition of his Essay on Yellow Fever. By Tho. Dancer, Author of the Jamaica Practice of Physic, or Medical Assistant.

Membranis intus positis delere licebit
Quod non edideris—Nescit vox missa reverti.

THE Essay on Yellow Fever, by Dr. Grant, published in 1802, has had ample justice done to it. The merits of that Work, both in point of composition and matter, were so conspicuous that they could not escape the notice of the *Reviewers*. In one Review it is designated as an *unique Production;* in an another it is *termed a non-descript in Literature;* another wicked Wight says, *that for any information it contains, the Author, who boasts of having practised in Jamaica for the space of* 40 *years, might as well have been all that time in Cheapside*; but why should the public be reminded of what is engraven in their memory, so as never to be forgotten.

What could possess this Writer, that he could not rest satisfied with the laurels he had gained in English composition, but that he must try his strength in Latin? What, but a pedantic ostentation, which has exposed him again to the rod of criticism? I shall therefore assume the task he has imposed on me, by this new and gross attack,* and display to the public the merits of this vaunted Translation, in which he pledged himself to his friends, to prove that he was an *elegant Classical Scholar,* and a *learned Physician,* and should any one venture to doubt it, or say he is a *Dunce,* he will take an Affidavit to the contrary, before the Mayor and the Corporation, to whom he has dedicated, though *perhaps without leave,* this new effort of his Genius.

What has induced him to give up his former Patron, His Royal Highness the Duke of Clarence? or whether the Duke of Clarence, ashamed of such a *Protégé,* has not given up him, I shall not take upon me to determine, but it is plain that he thought he had on this occasion a greater interest in the Corporation, and therefore dedicates his Work to the Hon. The Mayor, &c. Honorabili, &c. (see the publication.)

In

* Vide his Preface.

In fabricating this Dedication he seems to have derived considerable assistance from consulting some of the Inaugural Theses *pro gradu Doctoratus*, but he has after all made a miserable job of it.—He dedicates it *Honorabili*, &c. and then, without addressing them in the second person, *Viri Spectatissimi*, &c. he says, *Vestro Patrocinio committo. Vobis sum devinctissimus*.

Hoc Opusculum.—This insignificant Pamphlet is first offered as a token of Gratitude, for so I understand *Tributum parvum*, not the amount of his *Parish Tax*; next it is committed to the Patronage of the Corporation, Vestro Patrocinio committo. What patronage they may think it deserving I cannot tell; but I suppose the Author will never dedicate any thing to them again, if they do not record this *opus* in the *Archives of Regiodunum*.

Before quitting this rare Dedication, by which the Corporate Body may or may *not* think themselves flattered, I must observe, 1st, the Inconsistency of the Author, in asserting that the cure he recommended in Yellow Fever, was confirmed by the experience of 40 years, when he acknowledges that it commenced its ravages only in 1793; but this is not the only instance of self-contradiction to be met with in this Dealer in Assertion.

*Vides quid sit Hominis.*

Observe next the Compliment he pays *himself*, whilst he pays none to his *Patrons*. He has not, after the manner of Dedicators, bestowed one single virtue on any one Member of the Corporation, except their having conferred on him *immense* Benefits (large Fees), which he seems conscious he did not merit, though he says of himself, what no one will say for him, that for the space of 40 years he discharged the duties of a Physician creditably and diligently, *honeste et assidue*, though it is well known that during a great part of that time he practised as an Apothecary;* and since he has assumed to himself the character of a Physician, he has been in perpetual contention with other Medical Practitioners, constantly "engaged in the wild squabble of a wordy war."

*All things* being *duly* fulfilled on the part of the Author, and the task of the Printers, (which is a matter of great import-

* This would not be mentioned, but for the contempt he now entertains and expresses for this Order of the Profession.

importance)!!! being "*non perfunctorie sed vigilate et fideliter peractis,* most *accurately* and *faithfully performed,* the Opusculum *(parvi farrago libelli, barbarie et barbarissimis referti),* now makes its appearance, *Latine redditum.*

The matter of this Work has a much more serious claim to attention than the style. Dr. Grant's hobbling aukward Latin can do no harm to any one but himself; but the Doctrines he inculcates, if adopted, and the Practice he recommends, if followed, might be attended with the most mischievous consequences. It is right therefore that these should be primarily considered; but before I conclude, I shall not omit pointing out the elegancies in the Latin Diction, to convince him, that although I may not, (as he has formerly insinuated), be able to *translate* Latin, I know how to *write* it.

To make a shew of building his depletory Practice on some foundation, he has introduced some physiological and pathological observations on the blood, and the "*Constitutionum Conditiones,*" not in the former Edition. —These, he says, are two:—In the *first,* the blood from being in too great a quantity, overcomes the action of the vessels, and therefore it stagnates in them and is corrupted, "*Stat stagnat corrumpitur,*" *pa.* 11.

In the other, *Conditio Constitutionum,* the *compages* of the blood is destroyed and rendered putrid by a *too strong* action of the vessels.—If any admirer of this Pathology wishes to see more on the subject, let him consult *Boerhaave,* and his Commentator *Van Sweten, (De Obstructione.)* It is not the Pathology of the present day, I shall therefore pass it over, with all that he besides says, about the elementary parts and composition of the blood, observing only, that the appearances of blood when drawn are very fallacious, and in general throw little light on the nature of the disease present.—They are various under the same disease, and alike under many diseases of a very dissimilar nature. They can be even varied at will.

Upon this supposed *Stagnation of the blood in the vessels,* and of its *compages** *being mechanically broke down by the more forcible action of the vessels,* he founds his depletory Practice,

* *Compages* is certainly an improper word for expressing a chemical combination. It is a wonder, as the Doctor reads none but antiquated Authors, that he did not remember the term *Crasis.*

Practice, which, if he is to be credited, is next to infallible; but how contrary this is to truth, I need not repeat.

With respect to blood letting in Yellow Fever, I have to observe, that those who were formerly the most strenuous Advocates for this Practice have from experience of its fatal effects abandoned it. Dr. Jackson himself, the greatest champion for it, is forced to confess, that it can only be employed with safety in the very moment of time when the disease is forming. This is giving the matter up, for it is rarely that advice is applied for before the disease is already formed, and frequently it has made considerable progress.*

As to the use of Bark in Yellow Fever, *cane pejus & angue vitanda est,* I can aver that it is rarely it can be administered; and when administered, that it invariably does mischief. I appeal to the experience of the whole Profession (except of Dr. Grant), for the confirmation of this, as a fact; but Dr. Grant does not distinguish between Yellow Fever and the Endemial Remittent. The Yellow Fever, says he, is the *Remittens et Endemia Insulæ.* Whether it is to be considered as such, under an aggravated form, or whether it be a fever perfectly distinct, it is certain that it runs a very different course, as is manifest from the *symptomata Pathognomenica or Historia Morbi* † given by Dr. Grant himself; and he further observes, that the Yellow Fever differs from the *Causes* of the Ancients in this, viz. the *Causes* having regular exacerbations and remissions. The inference then is, that the Yellow Fever has not regular remissions and exacerbations, which is very true. Every one who has had opportunities of seeing Yellow Fever, knows that there is seldom more than one Paroxysm, which subsiding leaves the patient in such a state, that he fancies himself *well*, and his friends and medical attendants, who are not well acquainted with the insidious nature of these diseases, are liable to be imposed on by the favourable appearances.

---

* The greatest harm from the use of the lancet, appears to arise in those cases where it seemed most required. In proportion as the patient was full, robust, and vigorous, and the attack of fever severe, so in general was the rapid debility which followed. See Observations on Yellow Fever, in the Medical and Physical Journal.

† Let any one compare this confused account of the Symptoms in Yellow Fever, by Dr. Grant, mingled with Rules of Practice, to the clear elegant description in Dr. Charles M'Larty's Thesis pro Gradu Doctoratus, 1799.

ances. These however, are of short duration.—There is indeed no return of Fever; but a direful train of Symptoms, such as black vomit, hæmorrhagy, &c. coming on, the Patient sooner or later expires.

In Dr. Grant's description of the Yellow Fever, it is said that the Patient frequently in the beginning, vomits *pure bile.* This is what happens in the *ordinary remittent*, but rarely I believe in *real Yellow Fever*; and it is a circumstance that may serve among others, as a criterion in distinguishing the one fever from the other.

The Black Vomit, according to Doctor Grant, is so *acrid*, as to corrode the fauces, &c. "*Erravit spissum errorem.*" This is what I never saw nor heard of. Dr. Physic, of Philadelphia, in examining the matter of black vomit, was led to taste it, and gave it to animals in pills. It was found perfectly bland and innocent.

For some years past, viz. *since the Yellow Fever commenced its ravages,* Europeans of long standing, and even the Natives of the Island, have, (according to Dr. Grant) been subject to a fever of a more *aggravated* form, of a more *malignant* kind. This may be disputed; but admitting it may be true, what does it go to prove, but that there has existed since 1793, a fever differing widely from the *Endemia Remittens,* which Dr. Grant pretends to have been so successful in curing for forty years past. Further Dr. Grant says, that some Physicians *(inscii)* not acquainted like him with the *(Ratio Medendi)* with the method of treatment, contended that it was contagious.—Now, they might be ignorant of the manner of treating it, without supposing it contagious—but did not Dr. Grant himself, who pretended to know so much better (from forty years experience), incline with his friend Dr. Broughton, to the opinion that this fever was not the *Remittens Endemia,* but *Synochus?*—upon this point I shall urge nothing here, having imparted my sentiments at length in a paper inserted in the *Medical and Physical Journal,* Vol. XIV. as also in the *New-York Repository,* Vol. VII.

*Irritability of stomach,* (says Dr. Grant) often attends *even intermittent fevers,* but this direful symptom *(hoc dirum Symptoma)* in the Yellow Fever he verily believes *(quam verissime credit)* is occasioned by the unwarrantable practice of administering Jalap and Calomel in the exordium of the disease. If this be the case, what may not Dr. Grant have to answer for?—Search the Apothecaries files for his Prescriptions, and see if he never ordered these these *austere* Cathartics, which he at present reprobates and considers as the cause of this *(dirum symptoma)* direful symptom, viz. vomiting.

The Doctor's constant cry is *Canit eandem Cantilenam)*, that he is not called in time, not in the *Exordium*, and therefore his *successful* mode of treatment cannot *succeed*, and then he tell us how miraculously he *did succeed* in recovering patients, even after Hæmorrhagy and Black Vomit had come on. There is nobody but Dr. Grant that boasts of saving five Yellow Fever patients out of seven; but yet there are others that can exhibit cases fully as miraculous as those of Dr. Grant, where patients recovered under the Mercurial Treatment, which according to him, infallibly destroys every patient.—(See. before—*non unum convaluisse.)*

What Dr. Grant says on the subject of Mercury—of its stimulant power, and of its destroying the *Compages of the Blood, &c.* I shall leave for the consideration of more able Pathologists.—I shall only observe, that his notion of its tendency to produce putrescence is not confirmed by fact, for patients having taken several hundred grains of Calomel do not become *sooner* or *more putrid* after death, than those who have been treated in the *depletory* way, and who have used no Mercury in any shape whatsoever.

The information he pretends to have received, that the use of Mercury in the Naval and Military Hospitals is relinquished, is erroneous—he might on due enquiry have satisfied himself of the contrary; but this he did not wish, because he is willing to make it believed that the Surgeons in the Navy and Army are all proselytes to his bleeding or bloody system.

Formerly Dr. Grant insisted much on the appearances of the Brain on dissection; now, he says, *post mortem Dissectionibus non multum detegitur, quia Febris est habenda, totum systema amplectens et putre reddens.* Pray is not this the same fever that Dr. Grant had formerly considered as fatal, not from its occasioning a tendency to putrefaction, but from over distention and rupture of the blood vessels in the brain, to prevent which he so strenuously recommended the opening of the temporal artery, but which he never (according to what I have heard,) performed, *but in one instance?—Modo ait, modo negat, aliter dicit, aliter facit.*

Dr. Grant, the professed Enemy of all innovations in Medicine, (having practised forty years), has *heard* of the affusion of cold water in fever, which has been in use at at least for these ten years past, but of which *he knows nothing.*—*De cella frigidaria non mea experientia dicere possum*; but notwithstanding his acknowledged and blameable ignorance on the subject, he presumes to give cautions and directions about the use of the cella frigidaria, which is the term by which he has chosen to signify the use of cold water generally, viz. Affusion, Aspersions, &c. as well as cold bath.—*In Exordio* (says he) *durante inflammatorio statu haud voluissem.*—Now this period at which Dr. Grant *forbids* it, is exactly the one at which Dr. Currie, and after him, every one else, recommends it; —it is the only time for employing it with safety or advantage; but this does not accord with the Doctor's Pathology of *stagnation and corruption* of the Blood, and therefore he would not use it.

Lastly comes his advice to Tyros or newly arrived *Ensigns* and *Lieutenants,* for I know not how the appellation of Tyro is applicable to new comers in *Massa* (to use the Doctor's phrase).

Among other sage rules laid down to new comers for preserving health and guarding against Yellow Fever is this—viz. to wear flannel next the skin in the heat of the day, and to pull it off in the cold of the night; upon *what principle of Ratiocination* (to borrow his own language) he does this, I cannot comprehend. The Doctor cannot conclude without a little dabbling in Politics, for which he seems to have a great pruritus. If, says he, the Island of Jamaica would for a series of years *(per complures annos)* import annually *(quotannis,* a thousand troops, then *(brevi tempore)* in a *little time* (two or three years we will suppose) they would have a due supply, and would not be under the necessity of importing more.

I shall proceed no farther in my comments on Dr. Grant's *opusculum,* having nothing to do with the *Appendix,* which will, no doubt, be duly noticed by those who may think themselves concerned; and very *seriously concerned they are.*

Dr. Grant says he has erected a Temple to Truth;—say rather Monument, which will confer on him immortality.

---

Having pointed out the absurd Doctrines and dangerous Practice of Doctor Grant, in which he tenaciously persists in opposition to all the rest of the Profession, I shall now proceed to make some remarks on his Latin diction, diction, which I might have passed over, were I not assured that he plumes himself much on his proficiency as a Latin Scholar; and had he not been pleased to insinuate, that I was so ignorant as not to be able to translate two lines. It will not, therefore, be deemed presumption in me, should I contend with him for the Palm, and shew, that, notwithstanding he has been five years in conning this *Opusculum,* and that it bears so many marks of the file *(lima non defuit)*; his Latin is almost as bad as his English. It is constantly, or for the most part, either *vernacular* or *pedantic.* It either sinks into *downright* English or soars into *bombast.*—*Sophoclis Cothurnus.* It is either no Latin at all, or it is Latin scarcely to be understood.—It may be said of this Opusculum, *Vitiis abundat plurimis et solæcismis ubique scatet.*—

The first sentence in the Preface has a strong resemblance to the famous one in the English edition, that took a page and half, without being brought to a conclusion. —If it does not equal it in length, it does in obscurity; and may serve as a fit exercise to puzzle any one not used to such Latin as Dr. Grant's. [ See page 3. ]

Many more such examples might be produced; where the sense is obscure, if not unintelligible; and where the rules of Syntax are more or less violated.

It may be said, and justly, that it is easier to find fault with others than to excel ourselves: And it may be expected, that at the same time that *I point out* Dr. Grant's errors, I should *correct them!* —This I shall attempt to do in a few instances: But, to go through the whole would be an Augean Task.

Dr. Grant says, "*ut mea auctoritate scribo recentiora hac febre* NON PERSTRINXI.

*An intelligitur* ULLI?

☞ *Perstringere summatim, est breviter dicere.*—CICERO.

"CONTROVERSIIS ACORE EXUTIS." What kind of metaphor is this?

"PRIMA EDITIONE." *Lege, Editione priori.* There being at present only two: But, God knows, how many may follow!

"PRÆPROPERE FUIT PROMULGATA," Was hastily committed to the Press. To *promulgute* is to *proclaim* a law or odinance, not to publish a Book *Lege: Typis mandata fuit.*

"FACTA UT STANT,—As the facts stand." Cicero explains the meaning of the word *stare,* by saying: *Cui*

*opponitur sedere.* *Lege: Historiæ narratæ, narrationes citatæ.*

" Praxin deplendi promovent." *Lege, confirmant, stabiliunt.*

Argumenta *(sc. pro Hydrargii usu)* debilitant," weaken the argument! *Lege, Refellunt, confutant, faciunt ut minus valeant.*

" Corticem dispensandi—corticis dispensatione" *N. B.* To dispense a Medicine is to prepare and send it out, as an Apothecary does; not to prescribe, exhibit, or administer it.

" *Quædam digressiones politicæ prima editione* introducebantur." *Lege, inveniuntur.*

" Fædas stricturas," *sc.* Dr. Dancer's Strictures *non scintillæ ex ferro candente emissae non ejusmodi quæ opem poscunt chirurgicam—coarctatio nempe in urethra humana.*

*N. B Stricturæ* in Latin does not signify Criticism. But Dr. Grant being *perstrictus*, does not like *animadversiones.*

" Prima editione traditas." Does the Dr. mean here to inculpate himself of having in his first edition made any foul strictures. This is certainly what he says *(traditas)* and if he did not mean so to be understood, he is certainly chargeable with the fact: For, he has therein scandalously traduced and maligned the practitioners of Kingston.

" Nefariis promulgationibus," *viz.* The nefarious proclamations—not of the Corporation but of Dr. D.

" In massa *(in toto) quasi*, in a paste or a mass of Pills —and

" De authore viroso," *viz.* Dr. D. *tantum dicit: Kakou Korakos Kakon Oon—N. B.* Of Dr. Grant I shall say, *a maleficiis non temperabit, quotidie fit seipso deterior et sua ipsius nequitia se prodit—mens mala—mala lingua.*

" Chirurgis navium," To the Surgeons of Ships—much better than *Chirurgis nauticis—chirurgis artes suas in navibus exercentibus*—

" Hoc consilium," These directions: *lege, hæ monitiones, hæc monita vel præcepta.*

" Propitiore benevolentia recipiebantur," were more favourably received: *lege, benigniore animo accipiebantur, pluris æstimabantur.—Quia exitus ita secundus omnibus febribus non* arrideat." Magis mihi arrideat, evenit, accidit—

" *Multo minus Febri tam perniciosæ quam valde properæ & sæpe in ortu remisse vel ignave* consultæ." How is this this to be Englished? Does *consultare* signify *curam adhibere*: *Lege, advigilatæ* — "*Febris propera.*" This must be one of the *nova cohors febrium*, or does it signify *febris cujus cursus brevi perficitur?*

"*Calculo generali.*" Upon a general calculation. The Doctor is here quite out of his calculation. First, because *calculus* does not signify computation; and, secondly, he does not, as he asserts, save five Yellow Fever Patients out of seven. It may on the contrary be asked, *quot Themisson ægros autumno occiderit uno?*

"*Servavi.*" To pass over the egotism which occurs so frequently as to be disgusting. *Servare* does not signify to cure, or to restore to health. In the twentieth edition, the author may, if he pleases, substitute "*Saluti restituere, ad sanitatem, salvos reddere, morti abripere,*" *&c.* "*Exordium morbi.*" The beginning of the disease. Of the exordium of a book and of an oration, of the *primordium* of all things in the Universe, we have frequently heard: But whoever, before, heard of the *exordium* of a disease; but so partial is the learned Doctor to this sweet sounding word, that he never changes it for "*initium, principium, morbo adveniente, accedente, &c.* In the next edition I hope to hear of the *peroration* of the disease.

"Cura." The Doctor has the same liking to this word, which properly signifies *Care, not Cure,* and he will not on any account substitute *curatio, sanatio, medela, &c.*

"*Improvidus abusus.*" This was before found fault with as bad English. But the Doctor justifies it by putting it into *bad Latin.* He seems to take great delight in this species of pleonasm: For, speaking of sailors, he says, they are *inconsiderate improvidi.*—More examples of this elegant redundancy may be found—as *probra maligna, pertinaci pervicaciæ, propitiore benevolentia, opiata anodyna, &c.*

"*Durante morbo,*" during the whole course of the disease. *Lege, per totum morbi progressum aut decursum—quolibet morbi statu.*

*N. B. Durante morbo* signifies the disease continuing, *viz. non remittente.* But the discriminating Doctor makes the same blunder again, for he says, *durante navigio,* during the voyage, instead of saying *Inter navigandum, in transitu, in trajectu. Qu.* Does *navigium* signify a voyage? *Navigium est cujuscunque generis navis*—"*Veri pulveris Jacobi*" "True James's Powders." *Lege, Pulveris Jacobi fideliter præparati, non sophisticati.*

"*Absolute necesiaria.*" This wants no translating, not for country Gentlemen. *Lege, quam maxime vel planissime necessaria.*

"*In constitutionum modum novando.*" This is not translatable, unless perhaps it may mean altering the charter of the Corporation.—*Emeticorum prœpotentium.*" *Lege, immodicorum. Prœpotens* and *prœpollens* are terms suitable to persons, *viz.* Princes, Commanders; and *Emeticorum prœpotentium* might perhaps be translated the Emetics of the most puissant Buonaparte and the King of Prussia.

"*Austeris Catharticis*". *Lege, Catharticis fortioribus, vehementioribus, nimis efficacibus, hypercatharticis.*" *N. B.* This word austere applies to taste, and metaphorically to persons and manners, but not to Cathartics.

"*Post depletiones forsan salvo & commodo eventu* UTI POTEST *sc. balneum. Lege, Post depletiones commodum ac utile sit, felici vel secundo eventu adhiberi vel prœcepi possit.*

*Pilulœ ex Jalappa &c. consistentes.* This is not even Gallipot Latin. *Lege, compositœ, confectœ.*—"*Si remissio non apropinquet.*" *Lege, Febre non remittente, Remissione non accidente.*—UT *Clystere injiciatur,*—may be injected *As* or in *the way* of Clyster. *Lege, Ut fiat enema omni quadrihorio injiciendum.*

"*Externœ applicationes levare tendunt.*"—*Lege, Levamen adferunt, adjuvant multum ad hoc symptoma levandum aut sedandum conducunt—valde in hoc symptomate levando vel consopiando prosunt.* Why he says applications in the plural, I cannot tell, for he speaks but of one, *Cataplasm* being the nominative to another verb.

"*Ardua vitœ communia obvenire. Lege, arduis rebus occurrendis, arduis ut obveniendum sit, miseriis calamitatibusque vitœ avertendis aut sustinendis.*

"*Usus perseverans.*" *O caput pervicax atque perseverans!*—*Lege, Usus assiduus, non prœtermissus, per petuus, non interruptus.*

"*Attamen probare,*" But to prove. *Lege,* "*ut probem, ut plane convincam, ut plane appareat, ut evidens aut manifestum sit.*" *N. B.* This is a very ungrammatical way of using the infinitive mood. Again he says, to enjoy health, "*Salute frui,*" instead of saying, *aliquis ut salute fruatur, ad salutem bene conservandam ad sanitatem tuendam.*"

"*Tyronibus.*" *Lege, Europœis vel peregrinis nuper advectis, haud diu appulsis, recenter advenientibus, novitiis.*

"*Dormitorium,*" The sleeping apartment; but, perhaps, the patient cannot sleep: put him therefore, as well in another another chamber. *Camera, conclave, cubiculum, quo sine molestia decumbat.*

"*Navis oneris.*" *Lege, Navis mercatoriæ* vel *commercio occupatæ.*

"*Cursum Hydrargyri persistere suasit.*"

*Qu.* Does *persisto* govern the accusative case, or are we to suppose here an intended elegance, *quoad* being understood?

"*Constitutionum sunt duæ conditiones.*"

*Qu.* Does he mean to say that there are two kinds of ordinances in the Corporation, or that *Corporis humani duplex est conditio aut status?*

"*Die postero arcessebam.*" Is this a proof that the Doctor does not understand the difference between the active and passive verb; or does it shew his negligence, there being no correction of this mistake in the list of *Errata*, when all the literal blunders are noted and the commas put carefully to rights?

"*Hæ autem Regulæ non nimium cito contemnendæ.*" *Lege, Hæc consilia vel præcepta non derepente prætermittanda sunt.*

"*Ex litteris receptis.*" *Lege, acceptis.*

*Quis talia legendo a risu temperet?*

The learned Doctor, if he be diligent, may, perhaps, in *five years more*, find precedents to justify some of his Latin; but when it is considered how long he has been employed upon this *Opusculum jam exsibilatum*, we might have expected an *Opus ingens præclarum Maculis vacans;* but he was determined to write Latin, and depending as well *suo proprio Marte*, as *propria sua Auctoritate*, he wrote at all hazards, it is evident, *malle eum deficere quam desinere*.

Some of the Reviewers, who took notice of his *prima Editio*, observed, that if he MANAGED WELL he would, in a subsequent one, avail himself of the Remarks of his Antagonist.—How far he has availed himself of this advice they will see.

*Sine Rivali teque et tua Solus amares.*

